# Mineral Content and Extracellular Matrix Protein Expression in Mouse Growth Plates During Epiphyseal Fusion: An Observational Study

**DOI:** 10.1007/s00223-025-01391-9

**Published:** 2025-06-06

**Authors:** Xinhang Yu, Megumi Nakamura, Miyuki Mayanagi, Itaru Mizoguchi, Yasuyuki Sasano

**Affiliations:** 1 Division of Orthodontics and Dentofacial Orthopedics, Tohoku University Graduate School of Dentistry, Sendai, Japan; 2 Division of Craniofacial Development and Tissue Biology, Tohoku University Graduate School of Dentistry, 4-1 Seiryo-Machi, Aoba-Ku, Sendai, 980-8575 Japan

**Keywords:** Aging, Calcified cartilage, Epiphyseal fusion, Extracellular matrix, Growth plate, Scanning electron microscopy and energy-dispersive X-ray spectroscopy (SEM/EDS)

## Abstract

In humans, the growth plate cartilage is completely replaced by bone in late puberty, resulting in epiphyseal fusion. However, in rats and mice, commonly used experimental model systems, the growth plate does not fuse completely even after sexual maturation, making it difficult to elucidate mechanisms involved in epiphyseal fusion. In this study, we investigated age-related changes in the mouse growth plate to better understand the process of epiphyseal fusion. We used scanning electron microscopy and energy-dispersive X-ray spectroscopy (SEM/EDS) to examine the distributions and concentrations of minerals in the growth plate. In SEM images, the hypertrophic zone was observed as a bright area and other zones as dark areas at 10 weeks of age (W10). The bright area was further expanded at W55 than at W10. EDS analysis showed that P and Ca concentrations were high in this area, while C and O concentrations were low, indicating that the growth plate had calcified during aging. Alcian blue histochemistry revealed that the glycosaminoglycans of aggrecan were distributed in the growth plate at both W10 and W55. Immunohistochemistry showed that aggrecan and type II collagen were expressed throughout the growth plate at W10, but sparsely at W55. Type I collagen was expressed weak at both W10 and W55. Type X collagen and MMP-13 expression were observed in the hypertrophic zone at W10 but not at W55. This study demonstrated that although the mouse growth plate calcifies with age, it remains calcified cartilage for an extended period without being replaced by bone.

## Introduction

The thin layer of cartilage between the epiphyseal and metaphyseal bones at the end of long bones is referred to as the growth plate or the epiphyseal plate, and is where longitudinal bone growth occurs via endochondral ossification [1, 2]. The growth plate is composed of hyaline cartilage, which contains several predominant extracellular matrix (ECM) components including aggrecan, a large proteoglycan, as well as type II collagen [3]. It is divided into three major zones that correspond to the stage of chondrocyte differentiation, i.e., the resting, proliferative, and hypertrophic zones as ordered from the epiphyseal side [4]. In the resting zone, chondrocytes are irregularly scattered within the cartilage matrix and do not actively proliferate. In addition, it has been proposed that this zone contains numerous stem cell-like cells [5]. Chondrocytes in the other zones are arranged in columns that lie parallel to the long axis of the bone [4, 5]. In the proliferative zone, chondrocytes actively undergo cell division and proliferate longitudinally [2], while in the hypertrophic zone, chondrocytes increase in size until they are known as “hypertrophic chondrocytes” that are characterized by type X collagen expression [3]. The ECM surrounding the hypertrophic chondrocytes calcifies, and they eventually disappear [2]. Four possible mechanisms for the disappearance of chondrocytes have been hypothesized: apoptosis, autophagy, transdifferentiation, and hypoxia [6, 7].

In humans, the proliferation rate of chondrocytes in the growth plate gradually declines in late puberty, and completely ceases near its end. The process is known as growth plate senescence, and the mechanism responsible has not yet been fully elucidated [6]. Programmed senescence eventually leads the growth plate to become thinner and ultimately disappear as the cartilage is gradually and completely replaced by bone, a process called epiphyseal fusion or growth plate closure [7, 8]. Although epiphyseal fusion has conventionally thought to be the cause of the cessation of bone growth, some studies have suggested that bone growth ceases due to the termination of chondrocyte proliferation, consequently leading to epiphyseal fusion [9, 10]. However, in rats and mice—unlike humans—the growth plate does not completely fuse after sexual maturity under normal physiological conditions [11, 12]. This fact complicates our understanding of the mechanisms responsible for epiphyseal fusion and has made it difficult to elucidate them.

To investigate this phenomenon in greater detail, in this study we examined the concentration of minerals and the composition of structural proteins in the growth plate of young mice in which epiphyseal fusion had not yet occurred, as well as in aged mice in which epiphyseal fusion had occurred, albeit incompletely. We used scanning electron microscopy and energy-dispersive X-ray spectroscopy (SEM/EDS) to examine the distribution of minerals and their concentration within the growth plate. We also used immunohistochemistry to examine the expression of aggrecan and type II collagen, major components of the cartilage ECM, and type I collagen, the major component of the ECM of bone.

## Materials and Methods

### Experimental Animals

Male C57BL/6 mice at 10 and 55 weeks of age (W10 and W55) were used for all experiments. All mice were kept under specific-pathogen-free conditions under a 12 h light/dark cycle at a constant temperature of 23 ± 3 °C, and were given free access to a stock diet and water. All experimental procedures were reviewed and approved by the Institutional Laboratory Animal Care and Use Committee of Tohoku University.

### X-Ray Micro-computed Tomography (micro-CT)

Three mice each from the W10 and W55 groups were used for micro-CT analysis to investigate whether their growth plates had fused. Their knees, which contained an intact tibial growth plate, were then resected and fixed in 4% paraformaldehyde (PFA) in 0.1 mol/L phosphate-buffered saline (PBS), pH 7.4, at 4 °C overnight. Micro-CT images of fixed knee samples were obtained under standardized settings (i.e., 80 kV, 120 μA, 8 μm/pixel) using a Scan Xmate-E090 (Comscantecno Co. Ltd., Yokohama, Japan). Next, three-dimensional images of the knee were reconstructed using 3D slicer (https://www.slicer.org/), an open-source application.

### Bulk Sample Preparation for SEM

Six mice each from the W10 and W55 groups were used for SEM observation. Their tibias were fixed in 2% PFA and 2% glutaraldehyde in 0.1 mol/L PBS at 4 °C for 12–24 h. Fixed samples were then immersed in 20% sucrose solution overnight before being embedded in SCEM, a cryo-embedding medium (SECTION-LAB Co. Ltd., Yokohama, Japan), then frozen. Next, a Leica CM3505 S cryostat (Leica Biosystems, Nussloch, Germany) was used to trim the frozen samples to expose their proximal tibial growth plate, then to produce sections with a smooth surface for further observation. Finally, samples were dehydrated using a graded series of ethanol solutions and air-dried overnight.

### SEM/EDS for Elemental Analysis

Six bulk samples each at W10 and W55 were used to examine elemental concentration within the proximal growth plate. Growth plates without a conductive coating were then analyzed using a JSM-6390LA SEM equipped with an EDS detector (JEOL Ltd., Tokyo, Japan) in low-vacuum mode. Backscattered electron (BSE) images were obtained at an accelerating voltage of 15 kV and using a spot size of 60–70 at a working distance of 10 mm. Four lines (for line scan analysis) and ten points (for point analysis) were randomly selected from the BSE images of each sample, and the concentrations of phosphorus (P), calcium (Ca), carbon (C), and oxygen (O) were measured and then normalized to 100 atomic %. In addition, elemental maps were generated to visualize the distribution of P, Ca, and C in each sample, and overlay images of these three elemental maps were obtained.

### Hematoxylin and Eosin (H&E) Staining

Six mice at W10 and three mice at W55 were used to examine the histological structure of the growth plate. Their knees were resected and fixed in 4% PFA in 0.1 mol/L PBS (pH 7.4) at 4 °C overnight. Subsequently, mixed samples were decalcified in 10% ethylenediamine tetra acetic acid in 0.01 mol/L PBS at 4 °C before being dehydrated using a graded series of ethanol solutions, embedded in paraffin, and sectioned to a thickness of 5 µm. Some of the resulting sections were then stained with H&E.

### Immunohistochemistry

The sections close to those used for H&E staining of three mice each at W10 and W55 were selected for immunohistochemistry experiments to detect proteins in the matrix of the growth plate. First, sections for immunostaining of collagens were pretreated with 2.5% hyaluronidase sourced from bovine testes (Sigma, St. Louis, MO, USA) for 30 min at 37 °C. All sections, including those for the metalloproteinase (MMP)-13 immunoreaction immersed in 3% hydrogen peroxide in methanol solution for 15 min to inactivate endogenous peroxidase. Next, sections were treated with 5% normal goat serum (Fujifilm Wako Pure Chemical Corporation, Osaka, Japan) for 30 min before being incubated with rabbit antibodies against rat aggrecan (1:500, ab36861; Abcam, Cambridge, UK), mouse type I collagen (1:1000; ab21286; Abcam), human type II collagen (1:200; ab34712; Abcam), human type X collagen (1:200; 26984-1-AP; Proteintech, Rosemont, IL, USA), or human MMP-13 (1:200; ab39012; Abcam) for 2 h at room temperature. At the same time, negative control sections were incubated with 5% normal goat serum instead of a primary antibody. After washing with PBS, sections were incubated with a Histofine Simple Stain MAX PO (R) kit (Nichirei Co., Tokyo, Japan) for 30 min at room temperature. The resulting immunoreaction was then visualized using 3,3’-diaminobenzidine (DAB). Methyl green was used for counterstaining.

### Alcian Blue Histochemistry

The sections of three mice each at W10 and W55 were also used for Alcian blue staining to detect the glycosaminoglycans (GAGs) of aggrecan. Sections were stained with 1% Alcian blue 8GX (Merk KGaA, Darmstadt, Germany) in 3% acetic acid solution at pH 2.5 for 40 min then counterstained with 0.1% nuclear fast red for 3 min (Tokyo Chemical Industry Co., Ltd., Tokyo, Japan) at room temperature.

### Statistical Analysis

Statistical analyses were performed using SPSS 22.0 (IBM Corp., Armonk, NY, USA). The normality of the data obtained by SEM/EDS was assessed using Shapiro–Wilk tests, and we found that the data were not normally distributed. Therefore, we performed nonparametric Kruskal–Wallis tests followed by Dunn’s post-hoc tests to compare the three independent groups (*n* = 6). The threshold of statistical significance was set at 0.05.

## Results

### Calcification Assessment by Micro-CT

**Fig. 1:**
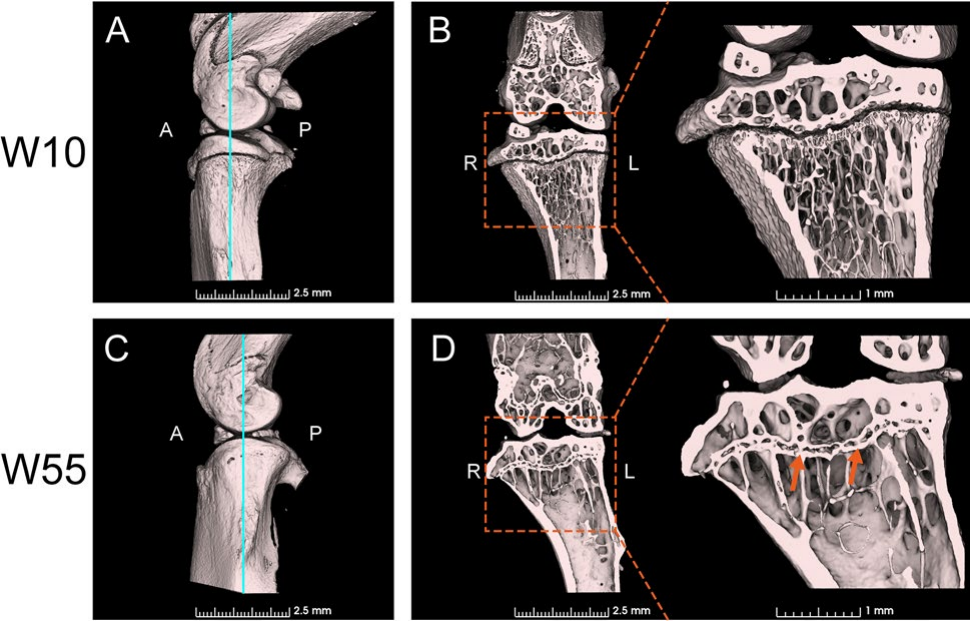
Three-dimensional micro-CT images of the knee and the proximal tibia. **A** The light blue line indicates the position of the coronal plane shown in B. **B** Coronal plane at W10. **C** The light blue line indicates the position of the coronal plane shown in D. **D** Sagittal plane at W55. Incomplete epiphyseal fusion is observed and several growth plate bridges are indicated by arrows. (A, anterior side; P, posterior side; R, right side; L, left side)

At W10, the proximal tibial growth plate was observed as a thin black plate-like region and was found to be unfused (Fig. 1A, B). In contrast, in W55 tibia, the growth plate was partially fused and calcified structures connecting the epiphyseal and the metaphyseal bones, referred to as growth plate bridges or bone bridges [13], were found within the growth plate (Fig. 1C, D).

### Examination of SEM Images of the Growth Plate

**Fig. 2:**
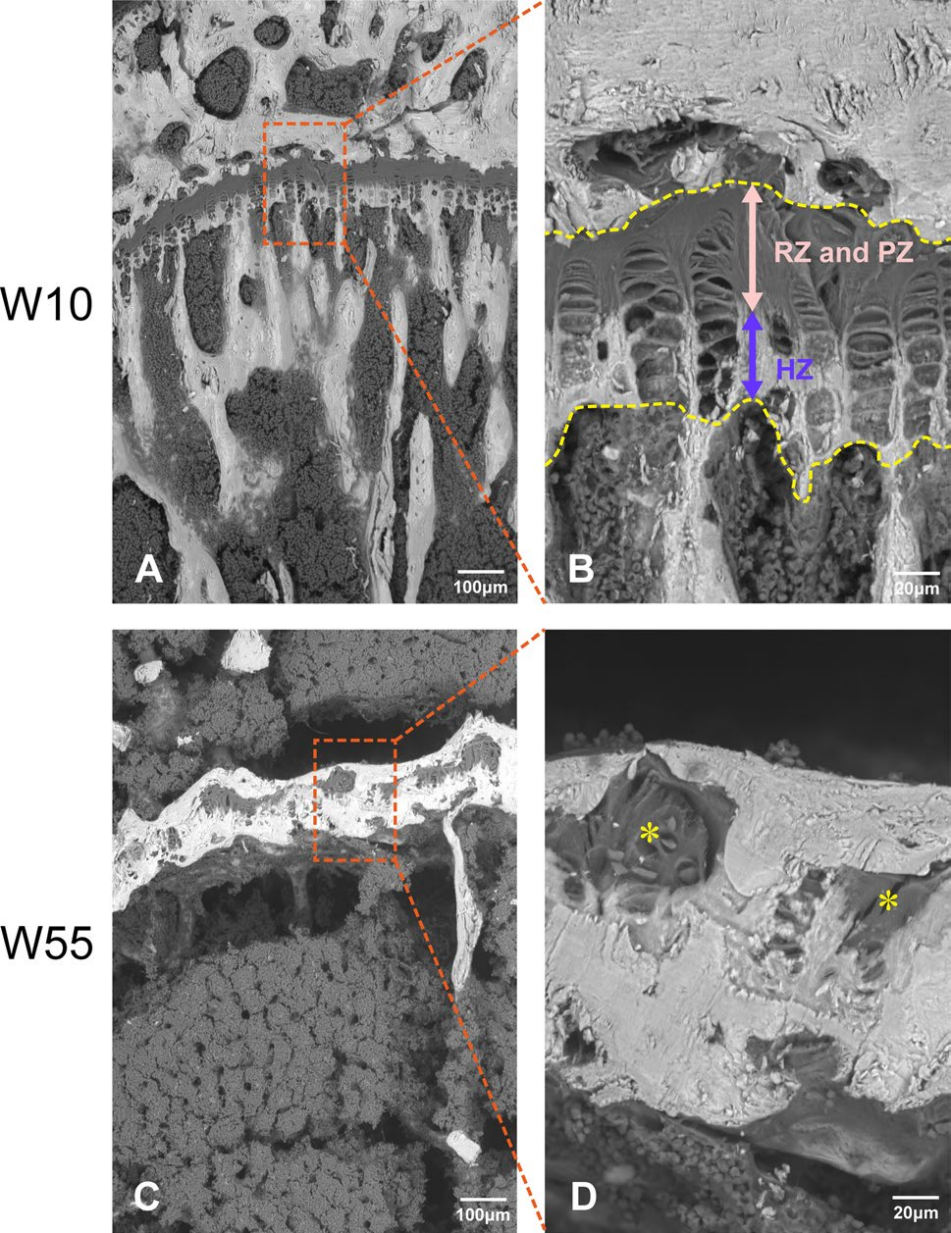
BSE images of the proximal end of the tibia as visualized by SEM.** A** Proximal tibial growth plate at W10.** B** Enlargement of the boxed area shown in A. This shows dark areas of the resting zone and proliferative zone as well as a bright area of the hypertrophic zone within the growth plate at W10. **C** Proximal tibial growth plate at W55. **D** Enlargement of the boxed area shown in C. Some islands within the dark area (*) can be observed within the bright area

Backscattered electron (BSE) images of the proximal tibial growth plate are shown in Fig. 2. For these images, the growth plate was identified as a thin band in which a dark zone was observed on the epiphyseal side and a bright zone on the metaphyseal side at W10 (Fig. 2A, B). Based on the morphology of chondrocytes or the chondrocyte lacunae aligned longitudinally within the growth plate, we determined that the dark area of the growth plate comprised the resting and the proliferative zones and the bright area as the hypertrophic zone (Fig. 2B). At W55, the bright area in the growth plate appeared to be more extensive than at W10, with some islands of the dark area present within the bright area. Moreover, the boundary between the epiphyseal or metaphyseal bone and the growth plate cartilage was not distinguishable in the bright area at W55 (Fig. 2C, D).

### Distribution of Minerals by EDS Elemental Mapping

**Fig. 3:**
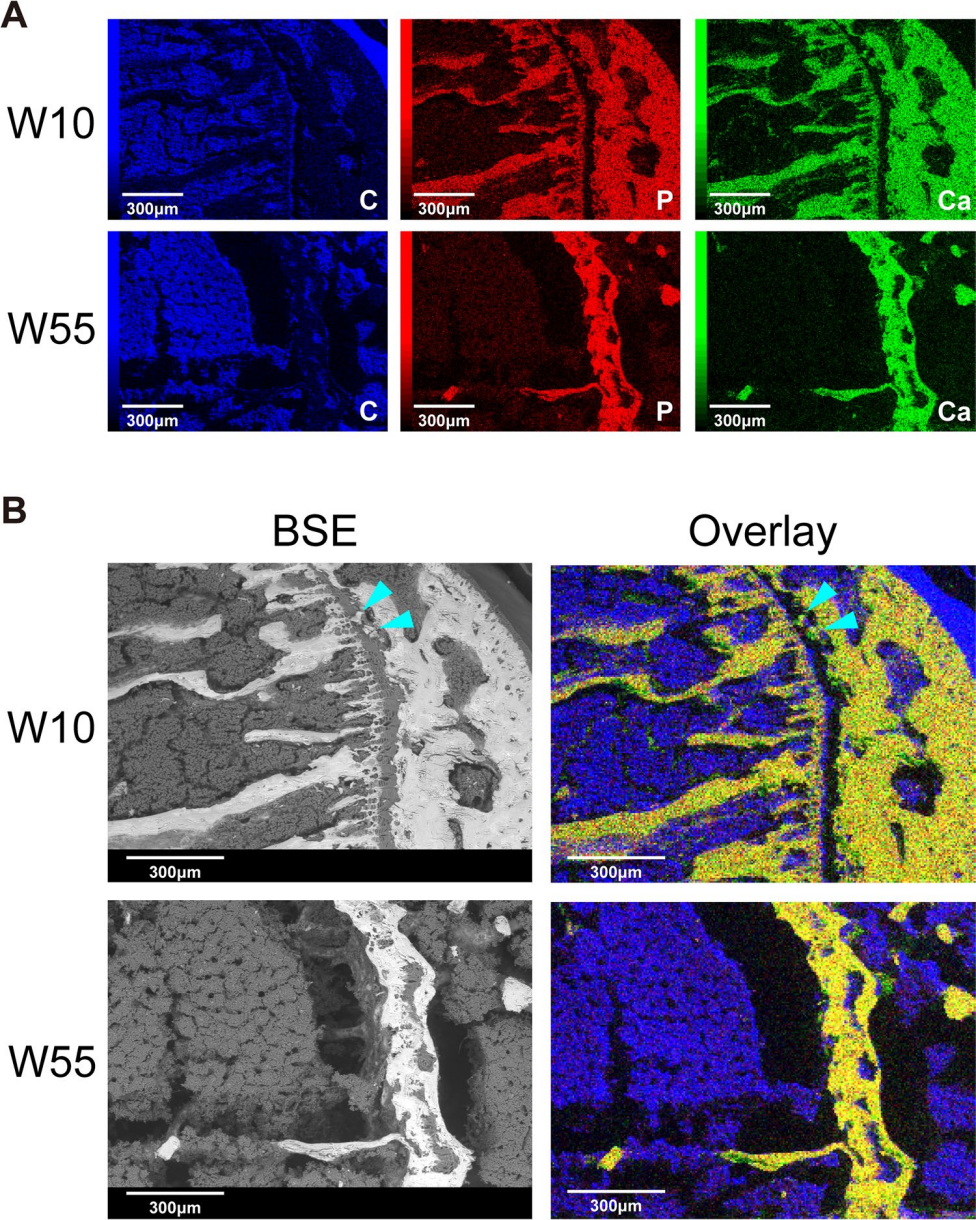
Elemental maps of the proximal tibial growth plate obtained via EDS. **A** Elemental maps of C (blue), P (red), and Ca (green) at W10 and W55. **B** BSE images and overlaid images of C, P, and Ca. Co-localized dots of P and Ca are shown as yellow in the overlay image. Scattered spots of mineral deposits are indicated by arrowheads

EDS elemental mapping based on BSE images was used to visualize the distribution of P, Ca, and C within the proximal tibial growth plate (Fig. 3). We found that P and Ca had not accumulated in the growth plate at W10, but both had accumulated in the growth plate at W55 (Fig. 3A). An overlay image of the three elemental maps showed that the distributions of P and Ca were consistent at both W10 and W55. The bright area from the BSE image corresponded to the mineral accumulation area on the overlay image. Interestingly, several scattered mineral deposits, which may represent the initial stage of bone bridge formation, were observed within the growth plate at W10 (Fig. 3B). Taken together, these findings indicate that the resting and proliferative zones of the growth plate were not calcified at W10, whereas they were partially calcified at W55.

### Quantitative Analysis of Mineral Concentrations

**Fig. 4:**
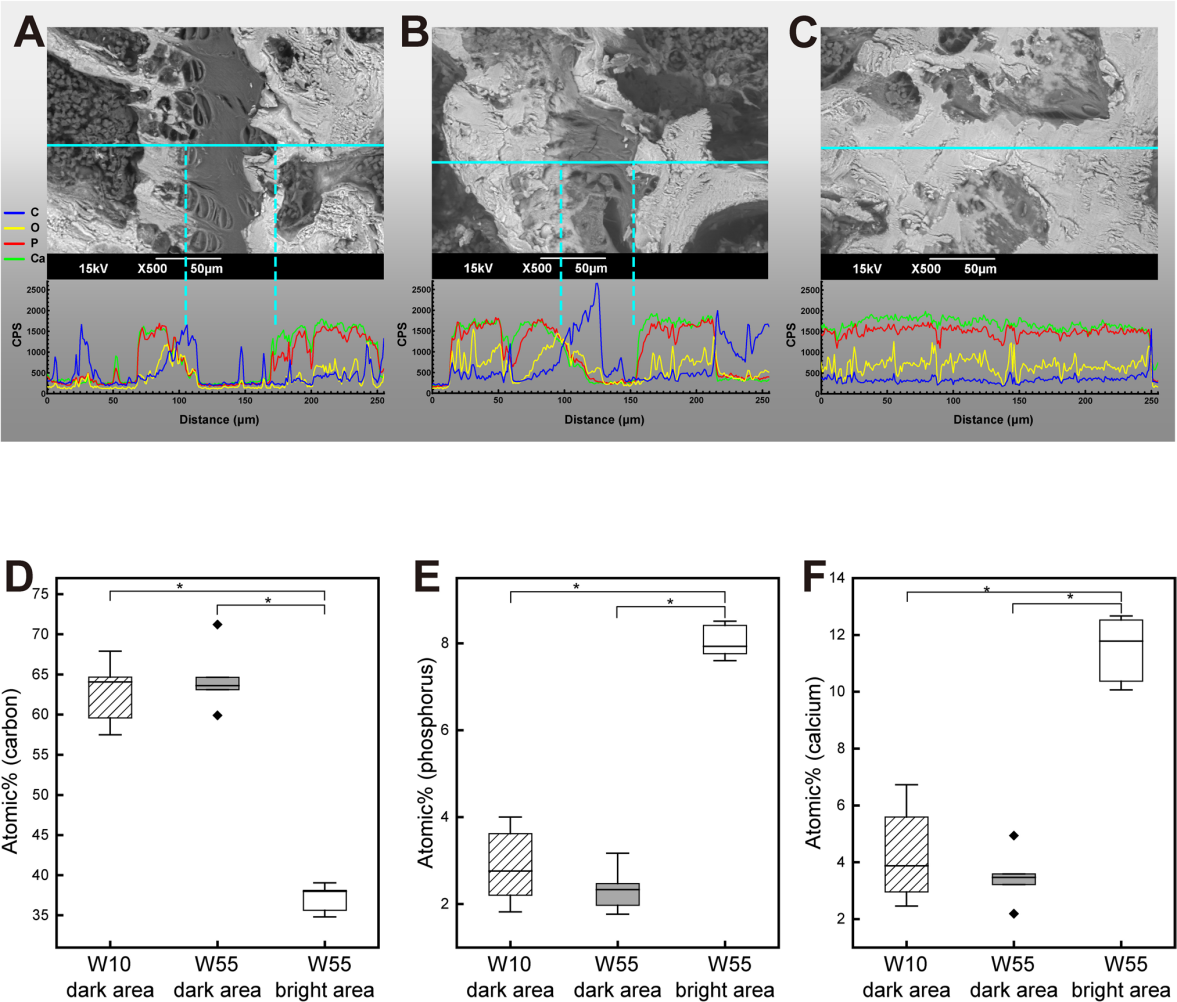
EDS line scans for C (blue) and O (yellow)—two main components of organic matter such as proteins, and P (red) and Ca (green), which are the main minerals in calcified tissues, along the light blue line on the BSE image. **A** Growth plate at W10. **B** Growth plate including the dark area at W55. **C** Growth plate excluding the dark area at W55. The concentrations of C, P, and Ca in the proximal tibial growth plate were measured via EDS point analysis. **D** The atomic percentage of C. **E** The atomic percentage of P. **F** The atomic percentage of Ca. (*n* = 6; Kruskal–Wallis tests with Dunn’s post-hoc tests; *P < 0.05)

Since elemental mapping results showed that the growth plate undergoes calcification during aging, we subsequently performed EDS line scan analysis to compare the mineral concentrations at different sites along the line. At W10, the concentrations of P and Ca were high in the epiphyseal bone and in the hypertrophic zone of the growth plate, but were low in the resting and proliferative zones of the growth plate. The concentrations of C and O were high around the boundary between the proliferative and hypertrophic zones of the growth plate, but were low at other sites (Fig. 4A). At W55, the concentrations of P and Ca were low and those of C and O were high in the dark area of the growth plate on the BSE image. Conversely, we also found that the concentrations of P and Ca were high and those of C and O were low in the bright area of the growth plate shown on the BSE image, including in the bone (Fig. 4B, C). Furthermore, we performed an EDS point analysis to compare elemental content in the dark area of the growth plate at W10 and W55 and in the bright area of the growth plate at W55. The concentration of C was significantly lower and those of P and Ca were significantly higher in the bright area at W55 than in the dark area at W10 or W55. There was no significant difference in the concentrations of all three elements between the dark area present at W10 and the dark area at W55 (Fig. 4D–F).

### Histological Characteristics of Growth Plate Chondrocytes

**Fig. 5:**
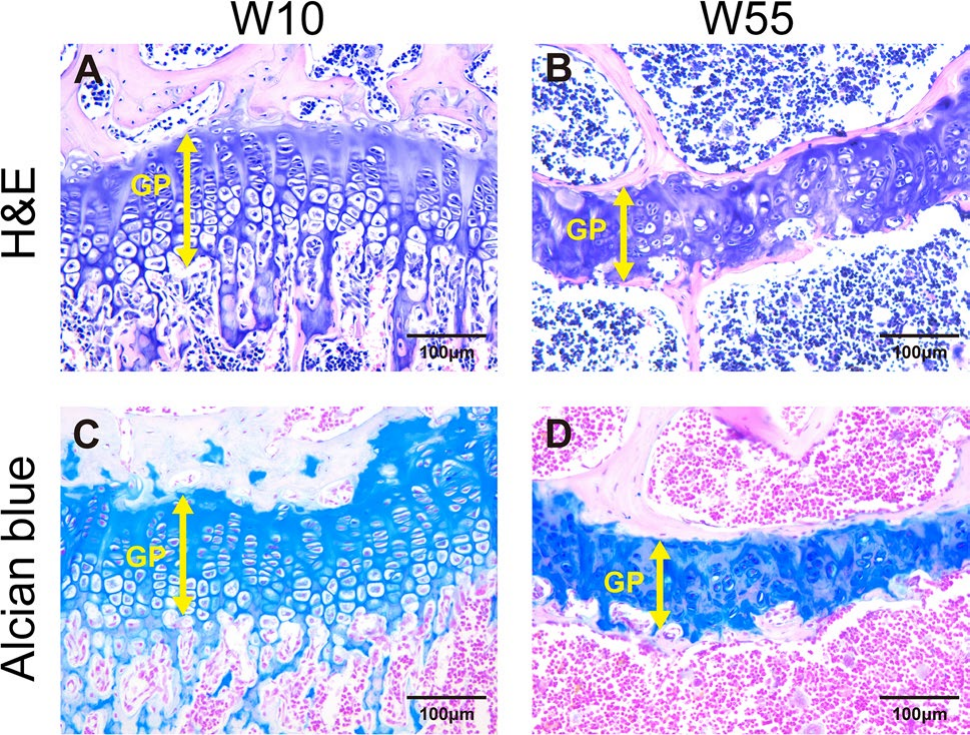
Sections of the proximal tibial growth plate stained with H&E or Alcian blue. **A** Growth plate stained with H&E at W10. **B** Growth plate stained with H&E at W55. **C** Growth plate stained with Alcian blue at W10. **D** Growth plate stained with Alcian blue at W55. *GP* growth plate

We used H&E staining for histological examination of the growth plate with aging. At W10, chondrocytes were arranged longitudinally in a single column, and these columns were regularly arranged in parallel along the growth plate. Three layers of the growth plate were observed—i.e., the resting, proliferative, and hypertrophic zones (Fig. 5A). At W55, in contrast, we observed that the thickness of the growth plate was thinner, the arrangement of chondrocytes was more irregular, and the cell density was lower. The individual zones of the growth plate were indistinguishable, and the chondrocytes within the aged growth plate had a unique morphology. Specifically, they were not as flat as the chondrocytes in the proliferative zone of the young growth plate, they were not as large as hypertrophic chondrocytes, and they had small nuclei and a bright cytoplasm. The histological examination revealed that no bone tissue was found within the growth plate at W55 or W10 (Fig. 5B).

### Distribution of GAGs, a Component of Aggrecan

Alcian blue histochemistry was performed to visualize the distribution of GAGs, a component of aggrecan, within the growth plate. At W10, the entire growth plate was stained with Alcian blue (Fig. 5C). At W55, even though Alcian blue was not detected in the epiphyseal and metaphyseal bones, the entire growth plate area between them was stained with Alcian blue, as it was at W10 (Fig. 5D). These findings indicate that the calcified region of the growth plate at W55 was not bone.

### Expression of Aggrecan, Type II Collagen, and Type I Collagen Proteins

**Fig. 6:**
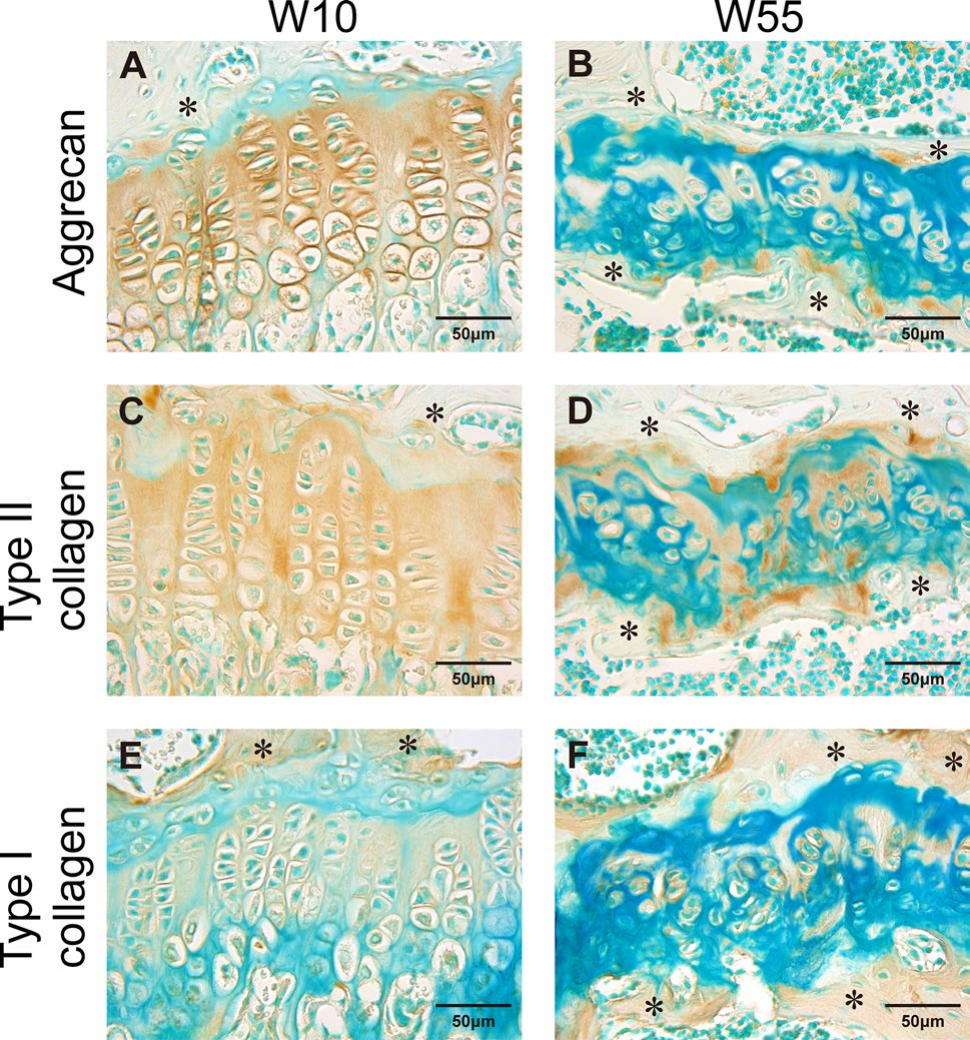
Sections of the proximal tibial growth plate following immunohistochemistry experiments. **A** Immunoreactivity of aggrecan at W10. **B** Immunoreactivity of aggrecan at W55. **C** Immunoreactivity of type II collagen at W10. **D** Immunoreactivity of type II collagen at W55. **E** Immunoreactivity of type I collagen at W10. **F** Immunoreactivity of type I collagen at W55. (*, bone)

We then examined the expression of the aggrecan and type II collagen proteins, two major components of the cartilage ECM, and type I collagen, the major component of bone ECM. At W10, immunoreactivity for aggrecan was observed throughout the growth plate, and strong immunoreactivity was observed within the pericellular matrix of chondrocytes (Fig. 6A). However, at W55, aggrecan immunoreactivity was not observed inside the growth plate, and strong immunoreactivity was observed only scattered along the border between the growth plate and the bone (Fig. 6B). As with aggrecan, immunoreactivity for type II collagen was seen throughout the growth plate at W10, but was only sparsely apparent in the growth plate at W55 (Fig. 6C, D). For type I collagen, immunoreactivity within the growth plate was very weak compared with surrounding bone at W10 (Fig. 6E). At W55, type I collagen immunoreactivity was localized only to the periphery of chondrocytes within the growth plate (Fig. 6F).

### Expression of MMP-13 and Type X Collagen Proteins

**Fig. 7:**
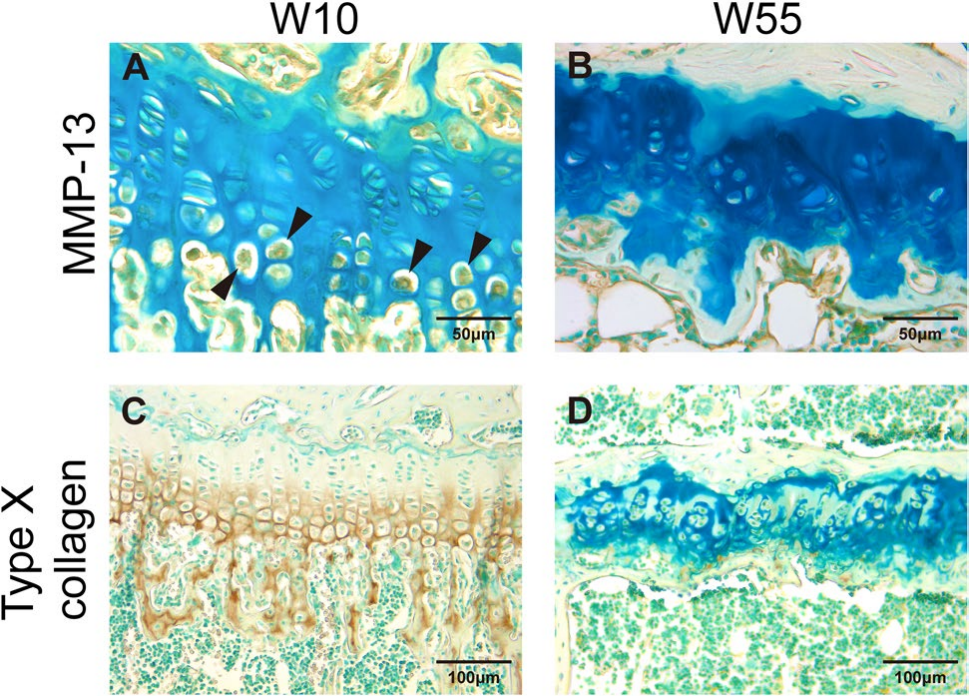
Sections of the proximal tibial growth plate following immunohistochemistry experiments. **A** Immunoreactivity of MMP-13 at W10. Hypertrophic chondrocytes expressing MMP-13 are indicated by arrowheads. **B** Immunoreactivity of MMP-13 at W55. **C** Immunoreactivity of type X collagen at W10. **D** Immunoreactivity of type X collagen at W55

We also examined the expression of MMP-13 and type X collagen proteins, two important markers of hypertrophic chondrocytes that are closely related to cartilage calcification. Immunoreactivity for MMP-13 was observed in hypertrophic chondrocytes at W10 (Fig. 7A), but not in the growth plate cartilage at W55 (Fig. 7B). Moreover, immunoreactivity of type X collagen was observed throughout the entire hypertrophic zone of the growth plate and partially in the trabecular bone at W10 (Fig. 7C). By W55, this signal was sparsely distributed around the margin of the growth plate on the bone marrow side (Fig. 7D).

## Discussion

This study demonstrated that the mouse growth plate calcifies during aging but also that it remains calcified cartilage for a long time without being replaced by bone. Evidence supporting this includes the facts that the growth plate at W55 was strongly stained for hematoxylin and was positive for Alcian blue, whereas bone was stained for eosin and negative for Alcian blue; therefore the W55 growth plate was determined to be cartilage, not bone. In addition, SEM/EDS analysis showed mineral accumulation within the growth plate at W55, indicating that the calcified tissue in the mouse growth plate at W55 was calcified cartilage instead of bone. In humans, invasive histological examinations have limited applicability, and noninvasive radiological analysis is the most common method used to investigate epiphyseal fusion. However, radiological approaches such as CT cannot determine whether calcified regions are comprised of bone or cartilage. Therefore, detailed histological findings on human epiphyseal fusion remain limited. Based on the results of this study, the human growth plate may also contain calcified cartilage for some period of time prior to replacement with bone.

In this study, the core protein of aggrecan was degraded at W55, while GAGs of aggrecan were accumulated within the growth plate at the same time point. Previous studies have shown that proteoglycans inhibit calcification in vitro [14–16]; however, GAGs have been reported to promote calcification. Wojtas et al. used an in vitro model based on mouse dental tissues to show that GAGs promote calcification in calcified tissue. In this experiment, the researchers prepared several GAG-removed groups using enzymatic treatment, a group in which non-collagenous protein was removed by trypsin treatment, and an untreated control group. The dental tissues of these groups were first demineralized, then remineralized using a calcium phosphate solution. Their results showed that the rate of mineralization in calcified tissues of GAG-removed groups was lower than the control group, and this effect was much greater than that of the non-collagenous protein-removal group [17]. Moreover, Purnomo et al*.* used exostosin-like glycosyltranferase 2-deficient (EXTL2 knockout) mice, in which GAGs were expressed at high levels in the aorta. They subsequently induced chronic kidney disease by feeding subject mice a high-phosphate diet, and found that matrix calcification was accelerated in aortic ring and vascular smooth muscle cells. They also showed that the removal of GAGs in vascular smooth muscle cells derived from EXTL2 knockout and wild-type mice effectively suppressed calcium deposition in a high-phosphate environment [18]. Therefore, GAGs that accumulate in the growth plate of aged mice may promote the formation of calcified cartilage.

We initially believed that growth plate cartilage would be replaced by bone due to aging, even in mice. Therefore, we predicted that immunohistochemical analyses would reveal a decrease in aggrecan and type II collagen, two major components of the cartilage matrix, as well as an increase in type I collagen, a major component of bone matrix, at W55. In fact, we observed reductions in aggrecan and type II collagen expression, as predicted, but type I collagen expression did not increase. This finding is one piece of evidence that mouse growth plates are not replaced by bone. Furthermore, an SEM/EDS elemental analysis revealed that the growth plate of aged mice was calcified. Accordingly, an additional immunohistochemical analysis was performed to examine the expression of type X collagen and MMP-13. Type X collagen is a non-fibrous collagen that constitutes the hypertrophic layer of cartilage prior to calcification, and hypertrophic chondrocytes express both type X and type II collagen [19]. In this study, type X collagen was expressed in hypertrophic chondrocytes and the hypertrophic layer of the growth plate cartilage at W10, but was hardly detected in the growth plate at W55. A previous study has reported that the synthesis of type X collagen ceases with the onset of cartilage calcification, followed by a decrease in its expression, and our findings are consistent with this explanation [20]. MMP-13 is an extracellular proteinase that cleaves type II collagen and aggrecan, and is expressed in hypertrophic chondrocytes [19, 21, 22]. It has also been suggested that type X collagen is degraded by MMP-13, and a previous report showed that MMP-13-deficient mice exhibited expansion of the hypertrophic zone of the growth plate and increased type X collagen deposition [23]. Previous studies have reported that MMP-13 may contribute to chondrocyte hypertrophy and cartilage matrix calcification via the degradation of type II and type X collagens and aggrecan [19, 24]. In this study, hypertrophic chondrocytes expressed MMP-13 at W10 but not at W55. Likewise, type X collagen was expressed throughout the hypertrophic layer at W10, but was hardly expressed at W55. Since the growth plate cartilage has already calcified at W55, MMP-13 degradation of type X collagen may be complete, and the synthesis of MMP-13 by chondrocytes may have already ceased.

Growth plate senescence leads to a decrease in the chondrocyte proliferation rate in the growth plate, reduced height (thickness) of the growth plate, and eventually a cessation of chondrocyte proliferation, resulting in epiphyseal fusion [9, 25, 26]. Interestingly, a previous study of growth plate transplantation in rabbits demonstrated that the growth rate of transplanted growth plates depended on the age of the donor, not the recipient [27]. This suggests that any reduction in the growth rate of the growth plate is caused by mechanisms intrinsic to the growth plate itself, rather than by the age of the individual or by factors in the environment external to the growth plate. In contrast, growth plate senescence has been shown to be independent of age, and is regulated chiefly by time—i.e., the cumulative number of chondrocyte divisions [28]. Furthermore, the proliferative capacity of stem cell-like cells within the resting zone has been reported to be finite; thus, growth plate senescence may be caused by the depletion of their proliferative capacity [29].

Estrogen is known to play an important role in longitudinal bone growth within the growth plate and is involved in epiphyseal fusion. For example, early estrogen exposure due to precocious puberty cause premature fusion of the growth plate, and can result in short stature [30]. Estrogen is an essential hormone for normal skeletal growth in both men and women. In a male patient who was estrogen-resistant due to a mutation in an estrogen receptor gene, no fusion of the growth plate was observed, and the patient continued to grow linearly beyond puberty to reach a height of 204 cm [31]. Estrogen can also promote epiphyseal fusion; however, the mechanism by which this occurs is not yet fully understood. A previous report using a female rabbit model showed that estrogen treatment of young, ovariectomized subjects resulted in accelerated growth plate senescence, including in a decreased proliferation rate of chondrocytes, reduced height of the growth plate, and fewer chondrocytes [9]. Thus, estrogen is considered to decrease chondrocyte proliferation and cause accelerated termination of their proliferation. However, the response of chondrocytes present in the growth plate to estrogen may differ between females and males. Moreover, the effect of estrogen on epiphyseal fusion may also differ among animal species. In this study, male mice were used for investigating epiphyseal fusion. This is in contrast to female rabbits, which were used in a previous report. Therefore certain hypotheses related to the mechanisms involved in epiphyseal fusion induced by estrogen may not be directly applicable to the male mouse model. To elucidate the mechanisms responsible for epiphyseal fusion, further investigations should be considered using different sexes and animal species.

Although in this study the growth plate was closed via calcification of growth plate cartilage, epiphyseal fusion generally means that the growth plate cartilage is replaced by bone in the final phase of growth plate senescence. The cartilage matrix of the growth plate is degraded, and chondrocytes disappear during epiphyseal fusion. The cellular mechanism by which chondrocytes disappear has not yet been elucidated, but four main hypotheses have been proposed: (1) apoptosis, (2) autophagy, (3) differentiation and conversion, and (4) hypoxia. A previous review has examined these four hypotheses in detail [6, 7]. The most widely accepted hypothesis is that hypertrophic chondrocytes undergo cell death by apoptosis. However, some previous studies have suggested that autophagy is involved in the death of hypertrophic chondrocytes [32, 33]. Another study by Emons et al*.* examined the disappearance of hypertrophic chondrocytes in the growth plate during the process of human epiphyseal fusion and reported no evidence of apoptosis or autophagy; in contrast, the authors reported morphological signs of hypoxia and necrosis [34]. The oldest hypothesis is that hypertrophic chondrocytes differentiate into osteoblasts within the growth plate [35], but to date this hypothesis has not been established. Further studies are therefore required to clarify the mechanism by which chondrocytes disappear during epiphyseal fusion.

In a previous study, Fukuda and Matsuoka radiologically studied the process from the appearance of secondary ossification centers to their fusion in the extremities of different animals. They found that patterns of epiphyseal fusion differed among animal species, among different types of long bones in the same animal, and even between the proximal and distal ends of the same long bone. These authors indicated that the incomplete epiphyseal fusion observed in mice and rats may be unique to certain rodent species, since it is not seen in humans, monkeys, or in dogs (beagles) [12]. Mice and rats are common model organisms used in life science and medical research; therefore, it is critical to thoroughly investigate their unique epiphyseal fusion processes. Furthermore, explorations of the involvement of epiphyseal fusion in diseases such as growth retardation and overgrowth may lead to new prevention and treatment strategies.

Epiphyseal fusion is generally considered to be a consequence of replacing growth plate cartilage with bone; however, this study demonstrated that the mouse growth plate calcifies during aging and remains as calcified cartilage, not bone, for a relatively long time. As mentioned above, the mice used in our experiments showed incomplete fusion of the growth plate, whereas complete fusion is observed in humans. Considering this, further studies are required to determine whether the mouse growth plate is replaced by bone after 55 weeks of age or is maintained as calcified cartilage thereafter, as well as whether calcified cartilage also appears both during and after epiphyseal fusion in humans.

## Conclusion

Our study revealed that the mouse growth plate calcifies during aging but also that it remains calcified cartilage for a long time without being replaced by bone, thus contributing to our understanding of the processes involved in mammalian epiphyseal fusion and providing new insight into the mechanisms of epiphyseal fusion.

## Data Availability

All data generated or analyzed during this study are included within the article. Further enquiries can be directed to the corresponding author.
